# Extending effective microwave absorbing bandwidth of CoNi bimetallic alloy derived from binary hydroxides

**DOI:** 10.1038/s41598-020-73161-6

**Published:** 2020-09-29

**Authors:** Weihua Gu, Jiabin Chen, Yue Zhao, Gehuan Wang, Fan Wang, Tengze Zhang, Baoshan Zhang

**Affiliations:** 1 School of Electronic Science and Engineering, Nanjing University, Nanjing, 210093 People’s Republic of China; 2 College of Material Science and Technology, Nanjing University of Aeronautics and Astronautics, Nanjing, 210016 People’s Republic of China

**Keywords:** Magnetic properties and materials, Nanoparticles

## Abstract

Effectively broadening microwave absorbing frequency of pure magnetic substances remains a huge challenge. Herein, micro-perspective structures can be controlled through a calcination route. Satisfactorily, the composites prepared at the calcination temperature of 900 °C exhibit excellent microwave attenuation performance with a broad working frequency and appropriate paraffin filling ratio. Remarkably, the composites can reach an extremely high reflection loss (RL) value of − 49.79 dB, and the extended effective working frequency range (RL < − 10 dB) of 6.84 GHz can also be obtained. Superb magnetic loss, admirable dielectric loss, sufficient dipole polarization, as well as superior impedance matching should be band together for obtaining ideal microwave absorbers. The CoNi hydroxides derived bimatallic alloy composites were fabricated via a cost-effective and facile synthesis process, and this work aroused inspirations of designing high-performance microwave absorbers for mataining the sustainable development.

## Introduction

Nowadays, rapid development of wireless communications, such as smart phones, portable computers and artificial intelligence, etc. has given rise to the spurting explosion of electromagnetic pollution^1,2^. In consideration of the sustainable development of the human beings, microwave absorbing materials with high-performance have aroused wide attention all over the world^3^. Due to the outstanding ferromagnetic characteristics, magnetic metallic materials such as Co, Ni and their alloys have unparalleled advantages among all kinds of candidates^4^.

Despite the high saturation magnetization and magnetocrystalline anisotropy, narrow effective bandwidth caused by inferior impedance matching has seriously limited their applications in practical situation^5,6^. In recent years, experts and scholars have hammered at broadening absorption bandwidth via introducing novel carbon-based materials, such as grapheme^7^, CNTs^8^, MXene^9^, etc. However, these kinds of raw materials are highly expensive, which have extremely impeded their industrial applications to some extent. Abundant researches on magnetic materials and their alloys have been reported to investigate the synthesis process and microwave absorption properties^10,11^. Take their high magnetic saturation, outstanding electrical conductivity and excellent temperature stabilization into account, magnetic materials and their alloys can be regarded as promising candidates for microwave absorption^12^. However, their stringent preparation conditions with complex and high-cost fabrication process limited the development of magnetic composites. Therefore, it is quite necessary to solve these problems. Bimetallic hydroxides as one kind of inorganic functional materials are attractive for extensive applications, such as supercapacitor^13^, catalyst^14^, and potentiometric sensors^15^, etc. Compared to conventional materials, bimetallic hydroxides materials have superior advantages of cost-effectiveness, chemical stability, environmentally nature, and so forth^16–18^. In this work, we take facile and cost-effective co-precipitation method to prepare the magnetic CoNi bimetallic hydroxides (CoNiBH). Fortunately, CoNi bimetallic hydroxide is beneficial to introduce cobalt and nickel positive ions, thus bringing about dual magnetic loss sources and large specific surface areas. Through controlling the heating temperatures, the CoNi bimetallic hydroxides derived products exhibited diverse morphology and size, which plays a significant role in achieving excellent impedance matching properties. Therefore, assembling appropriate ingredients via simple co-precipitation and tunable calcinations route is an effective strategy for extending microwave absorbing bandwidth.

Fortunately, the as-prepared CoNi bimetallic alloys derived from CoNi double hydroxides was obtained. More importantly, appropriate impedance matching performance and extremely strong electromagnetic attenuation capacity were achieved. When tuning annealing temperature to 900 °C, the RL_min_ value of − 49.79 dB appeared at the frequency of 15.68 GHz, and the broadest bandwidth was up to 6.84 GHz (11.16–18 GHz) with a thin layer thickness of 2.1 mm. The results not only demonstrated that the calcination process has great significance for forming suitable particle shapes with favorable impedance matching, but also provided a novel rational design inspiration into widening working frequency range for practical applications.

## Results

**Figure 1:**
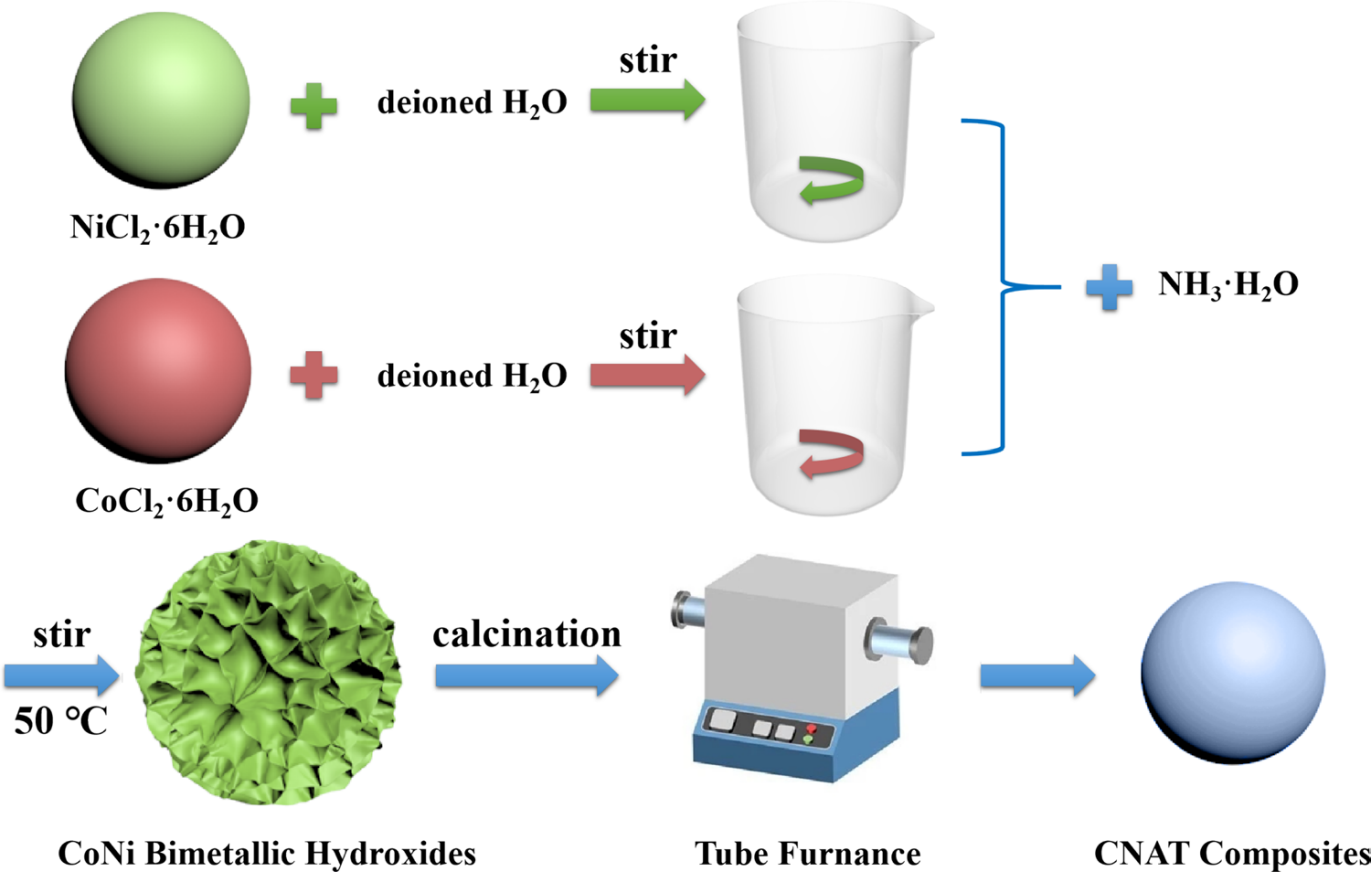
Schematic illustration of the synthesis process of CNAT hybrids samples.

**Figure 2:**
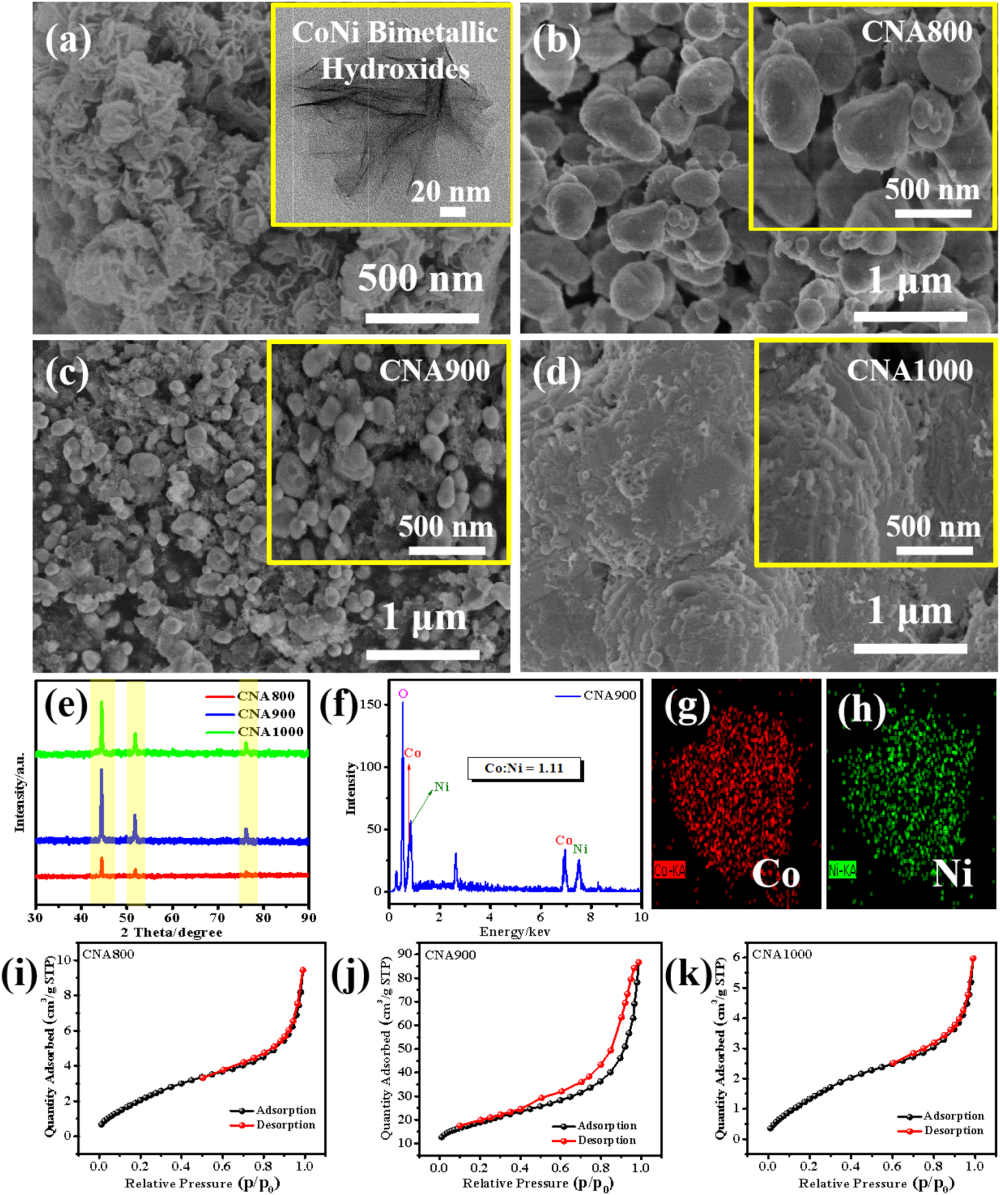
(**a**) SEM image of the CoNi bimetallic hydroxide precursors. Inset: TEM image of the as-prepared precursor. SEM images of (**b**) CNA800, (**c**) CNA900 and (**d**) CNA1000. (**e**) XRD pattern of CNA700/800/900. (**f**) EDS of CNA900 and (**g**,**h**) element EDS mapping of CNA900. The N_2_ adsorption–desorption isotherms of (**i**) CNA800, (**j**) CNA900 and (**k**) CNA1000, respectively.

The facile synthesis process of nanoflower-like bimetallic hydroxides is depicted in Fig. 1. Obviously, a series of typical calcinations treatment are carried out to fabricate CoNi alloy composites. The formation of CoNi alloys can be ascribed to two possible factors: (1) The residual ethanol caused by centrifugal washing and blast air drying process may bring a small amount of carbon source, thus, metallic oxides can be reduced to metals; (2) the residual NH_4_Cl may produce NH_3_ (gas) and HCl (gas) at a high temperature under the nitrogen atmosphere, herein, metallic oxides can also be reduced to metals. The relevant reaction process can be described as the following equations^19,20^:

1 $$ {\text{NH}}_{{4}} {\text{Cl }} \to {\text{ NH}}_{{3}} \left( {\text{g}} \right) \, + {\text{ HCl }}\left( {\text{g}} \right) $$

2 $$ {\text{M}}\left( {{\text{OH}}} \right)_{{2}} + {\text{ calcination }} \to {\text{ MO }} + {\text{ H}}_{{2}} {\text{O }}\left( {\text{g}} \right) $$

3 $$ {\text{2MO }} + {\text{ C }} + {\text{ calcination }} \to {\text{ 2M }} + {\text{ CO}}_{{2}} \left( {\text{g}} \right) $$

4 $$ {\text{3MO }} + {\text{ 2NH}}_{{3}} + {\text{ calcination }} \to {\text{ 3M }} + {\text{ N}}_{{2}} \left( {\text{g}} \right) \, + {\text{ 3H}}_{{2}} {\text{O }}\left( {\text{g}} \right) $$

where M(OH)_2_ means CoNi bimetallic hydroxides, MO stands for CoNi oxides and M signifies CoNi alloys. The morphologies of the CoNi bimetallic hydroxide precursors and final CNAT composites were systematically characterized, which can be seen from Fig. 2a–d. As described in Fig. 2a, flower-like hydroxides precursors with nanosheets intersecting morphology was fabricated initially via co-precipitation. With the increase of annealing temperature, one can find that the size of the final product exhibits an inverted “V” trend. For CNA800 and CNA900, the microstructure remains the form of nanoparticles. However, for CNA1000, particles aggregated together to a block shape, which may result in impedance mismatch. As provided in Fig. 2e, the typical diffraction peaks located between 30° and 90° can be well indexed to the (111), (200) and (220) planes of CoNi alloy, demonstrating that the above reaction equations are of high credibility level. Besides, all the peak positions are consistent with either metal Co (JCPDS No. 89-4301) or metal Ni (JCPDS No. 89-7128). Furthermore, intensity of the XRD patterns demonstrates the high purity and crystallinity of the CNAT composites. At the same time, the energy dispersive X-ray spectrometry image and element mapping results of CNA900 can be observed in Fig. 2f–h. It should be noticed that the atom ratio of Co and Ni in Fig. 2f is calculated to be 1.11, which is close to the ingredient ratio of the added raw materials, indicating that CoNi bimetallic particles have been successfully manufactured. Equally important, cobalt and nickel elements are marked by red dots and green dots in Fig. 2g,h, indicating homogeneous distribution of the two metal components. With regard to all samples, the smaller nanoparticles are presented, the more specific surface area is obtained. As shown in Fig. 2i–k, the BET surface areas of CNA800/900/1000 samples are 9.28, 67.04 and 6.50 m^2^/g, respectively, indicating that the CNA900 possesses the highest specific surface area. Therefore, CNA900 possesses more contact interfaces as well as superior electron transport channels. In a word, it can be concluded that only by adopting the appropriate annealing temperature, well-sized CNAT composites could be preferably synthesized.

## Discussion

**Figure 3:**
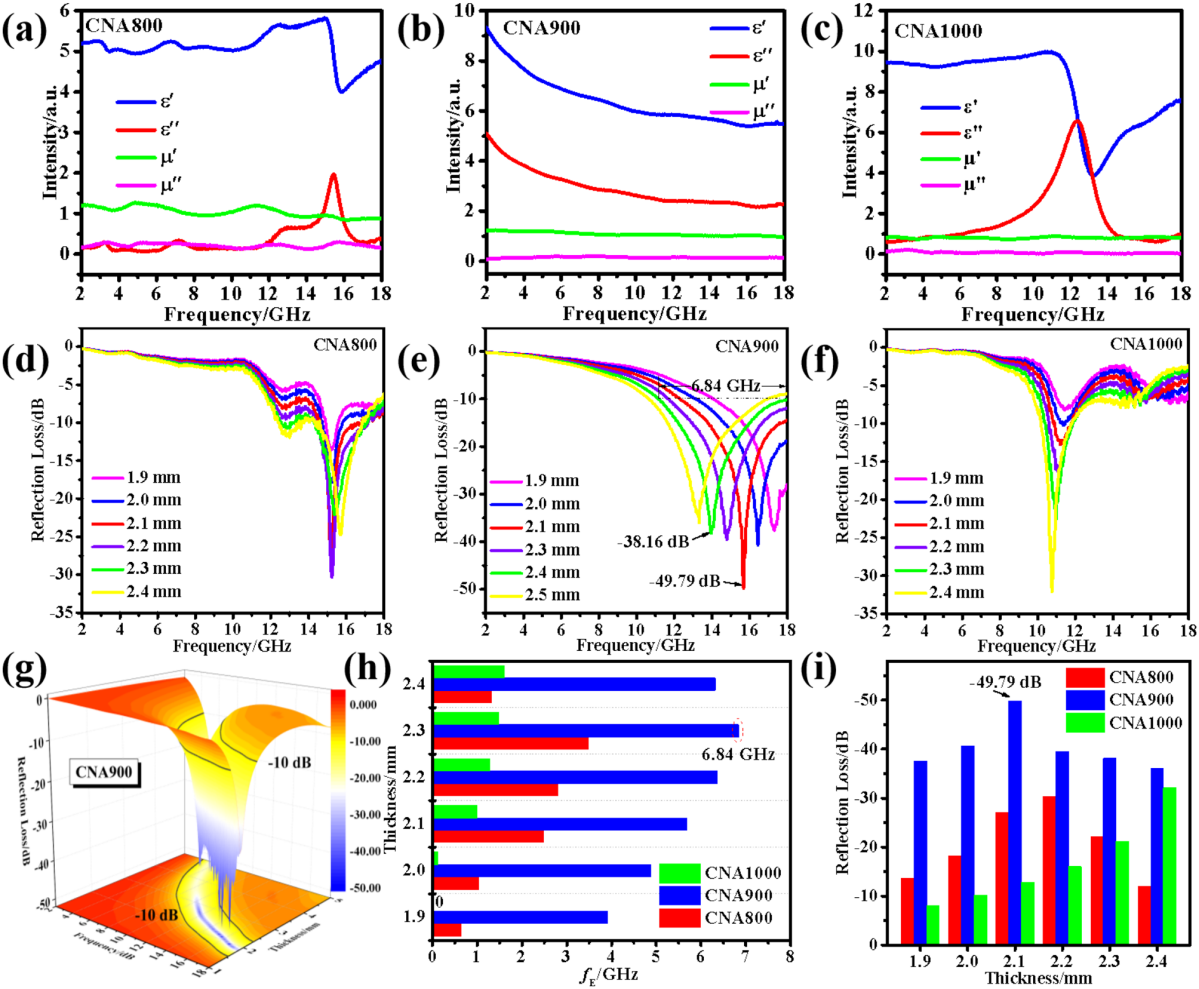
The electromagnetic parameters values of (**a**) CNA800, (**b**) CNA900 and (**c**) CNA1000 samples. Reflection loss values versus frequency of (**d**) CNA800, (**e**) CNA900 and (**f**) CNA1000. (**g**) 3D RL plots of CNA900. (**e**) Effective bandwidth and (**f**) minimum reflection loss values at different thicknesses, respectively.

Figure 3a–c exhibit the electromagnetic parameters of CNA800/900/1000 samples, including *ε′*, *ε″*, *μ′*, and *μ″,* respectively, which represent the real and imaginary parts of permittivity and permeability. As a rule, the real parts represent the storage performance of electrical and magnetic energy, while the imaginary parts signify the dissipation capacity of microwave energy^21^. CNA800 exhibits the lowest relative electromagnetic parameters among all the as-prepared composites with the same filling ratio. At the same time, the *ε′* value of the CNA900 sample decreased from 9.32 to 5.47 during the working frequency from 2 to 18 GHz, while the *ε″* value decreased from 5.10 to 2.25. It can be found that the *ε′* and *ε″* values of CNA800 and CNA1000 accompanied with dramatic fluctuations during high frequency range, which can be ascribed to dielectric resonance behavior and dipole polarization caused by particles aggregation^22^. Moreover, the average values of the real (*μ′*) and imaginary (*μ″*) permeability of CNA900 are 1.08 and 0.15, separately. In addition, the *μ′* and *μ″* values of CNA800 and CNA1000 specimens keep around at 1 and 0, respectively, which are similar to air. Complex permeability *μ*_*r*_ values maintain constant during the working frequency, indicating high stability of the magnetic dissipation capacity of the as-made samples. Based on the free electron theory, *ε″* = *1/πε*_*0*_*ρf*^23^, where *ρ* signifies the electrical resistivity, higher electron conductivity gives rise to larger imaginary parts of permittivity. Namely, CNA900 possesses the strongest dielectric dissipation capacity among all CNAT samples, which may further promote the microwave attenuation behaviors. For the purpose of evaluating the microwave absorption performance of the as-synthesized CNAT composites, reflection loss values (RL) curves are shown in Fig. 3d–f according to the basis of generalized transmission line theory and metal back model^24^:

5 $$ Z_{in} = \, Z_{0} \left( {\mu_{r} /\varepsilon_{r} } \right)^{1/2} tanh[j\left( {2\pi fd/c} \right)\left( {\mu_{r} \varepsilon_{r} } \right)^{1/2} ] $$

6 $$ RL \, = \, 20log\left| {\left( {Z_{in} - Z_{0} } \right)/\left( {Z_{in} - Z_{0} } \right)} \right| $$

Herein, *Z*_*in*_, *Z*_*0*_, *μ*_*r*_, *ε*_*r*_, *f*, *d*, and *c* stand for the input impedance value of the absorber, the impedance value of free space, complex permeability, complex permittivity, the whole measuring frequency, the thickness of the microwave absorber coating layer and the velocity of light, respectively. Obviously, when the coating thickness varies from 1.9 to 2.4 mm, RL values below − 10 dB could be achieved by all samples, which is appropriate for practical application (90% effective microwave absorption)^25^. As for CNA800 and CNA1000, the values of RL_min_ are − 30.33 dB and − 32.11 dB, separately. Evidently, the 3D representation of the RL performance of CNA900 is directly exhibited in Fig. 3g and the black bold lines stands for the value of − 10 dB. It should be noted that the RL_min_ value of CNA900 is − 49.79 dB at 15.68 GHz with 2.1 mm (Fig. 3e,i). In order to intuitively compare the microwave attenuation capacity of the as-prepared samples, the effective bandwidth and RL values at thin matching thickness are shown in Fig. 3h,i. In detail, the effective working frequency of CNA800 at different thicknesses are all less than 2 GHz, and the largest bandwidth of CNA1000 is less than 4 GHz. Compared with CNA800 and CNA1000, CNA900 exhibits an extremely broad effective bandwidth of 6.84 GHz (from 11.16 to 18 GHz) at a thin layer thickness of 2.3 mm. On the contrary, CNA800 and CNA1000 show narrower band range in the full working frequency, which can be caused by inferior impedance matching and unsuitable microscopic dimensions and morphologies. Therefore, the outstanding performance brings CNA900 a bright prospect for practical applications as ideal electromagnetic absorbers with strong absorbing properties, thin coating layer thickness and broad bandwidth.

**Figure 4:**
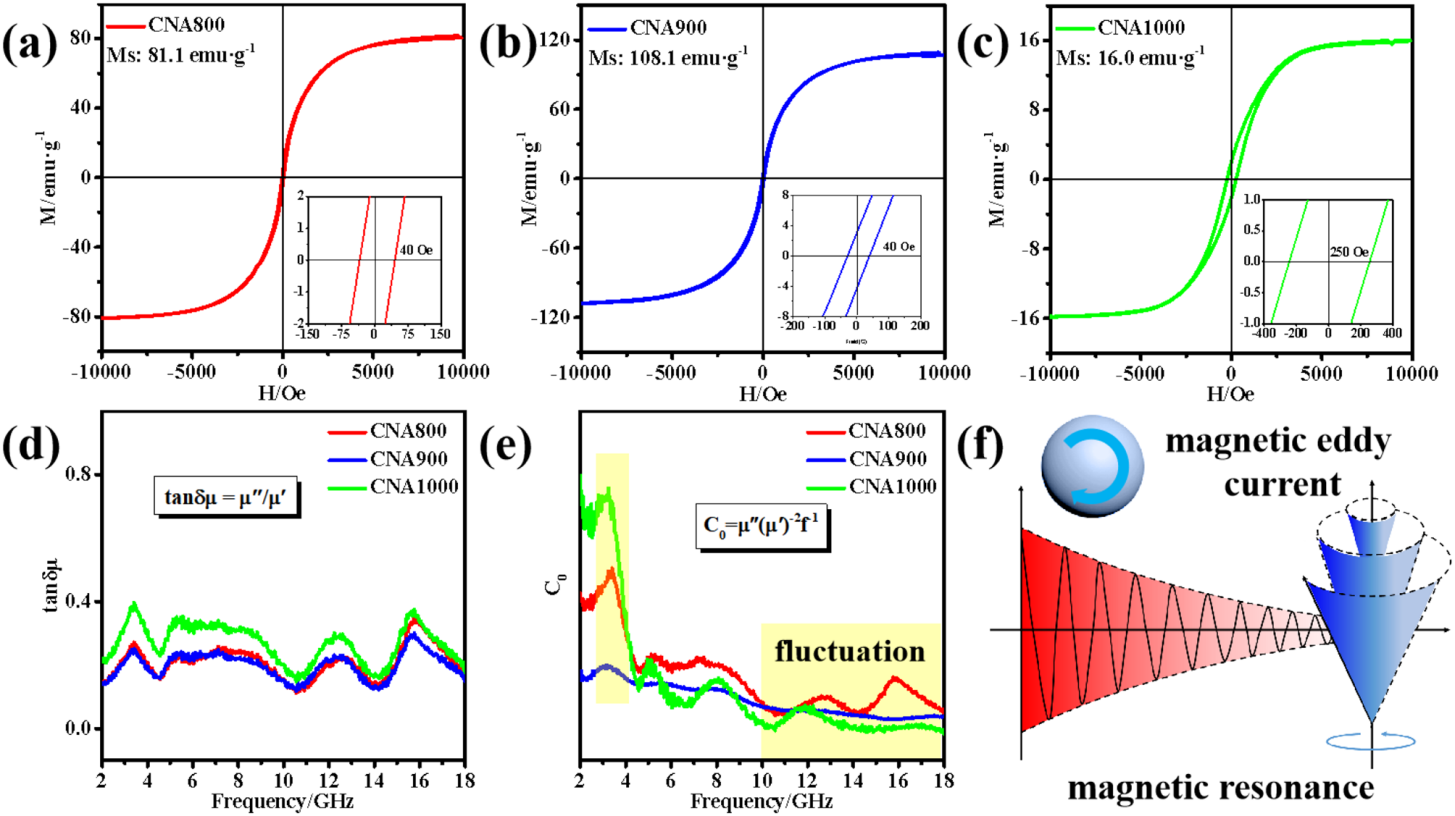
Hysteresis loops of (**a**) CNA800, (**b**) CNA900 and (**c**) CNA1000. (**d**) Magnetic loss tangents and (**e**) *C*_*0*_ values of the as-prepared samples. (**f**) Schematic illustrations of magnetic loss mechanism in CNAT composites.

In consideration of the presence of Co and Ni magnetic ingredients of the microwave absorbing materials, magnetic loss mechanisms have been provided in Fig. 4. As depicted in Fig. 4a–c, the hysteresis loops with relatively thin S-shape indicates that the CNAT composites exhibit soft magnetic characteristics. The saturated magnetization values (*M*_*s*_) of CNA800/900/1000 are 81.1, 108.1 and 16.0 emu g^−1^, respectively. Based on the *μ-M* equation^26^:

7 $$ \mu \prime \, = 1 \, + \left( {M/H} \right) \, \cos \sigma $$

8 $$ \mu \prime \prime \, = 1 \, + \left( {M/H} \right) \, \sin \sigma $$

where *M* is the magnetization, *H* represents the intensity of external magnetic field and *σ* signifies the phase lag angle of magnetization behind applied magnetic field. It can be deduced that higher *M* value result in higher complex permeability *μ*_*r*_ values. Besides, the size shrinkage in CNA900 may lead to more magnetic dissipation behaviors, which may result in augment values of *M*_*s*_. Furthermore, the coercivity values (*Hc*) of the as-prepared CNA800/900/1000 composites are 40 Oe, 40 Oe and 250 Oe, respectively. The higher *Hc* value (250 Oe) is related to the larger shape and surface anisotropy of the CNA1000 sample^27,28^. As shown in Fig. 4d, the average values of magnetic loss tangents (*tan δ*_*μ*_ = *μ″/μ′*) for CNA800/900/1000 are 0.20, 0.21 and 0.27, respectively, indicating similar magnetic loss ability of all as-prepared samples. As a rule, domain wall resonance usually exists in MHz frequency and hysteresis loss usually can be ignored in weak electromagnetic field. Therefore, magnetic attenuation mechanisms can be closely related to not only eddy current loss, but also natural resonance and exchange resonance^29^. Typically, eddy current loss (*C*_*0*_) can be assessed based on the following formula^30^:

9 $$ C_{0} = \mu \prime \prime \left( {\mu ^{\prime}} \right)^{ - 2} f^{ - 1} = \, 2\pi \mu_{0} d^{2} \sigma $$

Herein, *μ*_*0*_, *d*, and *σ* signify the permeability in vacuum, thickness and electrical conductivity, separately. If the eddy current loss is the dominating reason in magnetic loss mechanism, the *C*_*0*_-*f* curve will present a horizontal line during the testing frequency. With regard to CNA900, the *C*_*0*_ values almost keep a constant at high frequency range, demonstrating the positive effect of eddy current loss (Fig. 4e). However, for CNA800 and CNA1000, the *C*_*0*_ values fluctuate a lot, so the eddy current loss is not the main reason for magnetic attenuation. However, it still works to some extent. According to the natural resonance equation, *2πf*_*γ*_ = *γH*_*k*_^31,32^, where *f*_*γ,*_ means the natural resonance frequency, *γ* is electronic gyro-magnetic ration and *H*_*k*_ stands for the magneto-crystalline anisotropy field. Generally, the natural resonance occurs in low frequency range (0.1–10 GHz), an obvious peak at around 3.23 GHz can be seen from the highlighted part in Fig. 4e. Besides, exchange resonance often appears at high frequency part. Furthermore, schematic diagrams of magnetic eddy current loss and magnetic resonance are exhibited in Fig. 4f. Consequently, relevant magnetic loss mechanisms, such as eddy current loss, natural resonance and exchange resonance, correspond with relative permeability characteristics and contribute to microwave attenuation.

**Figure 5:**
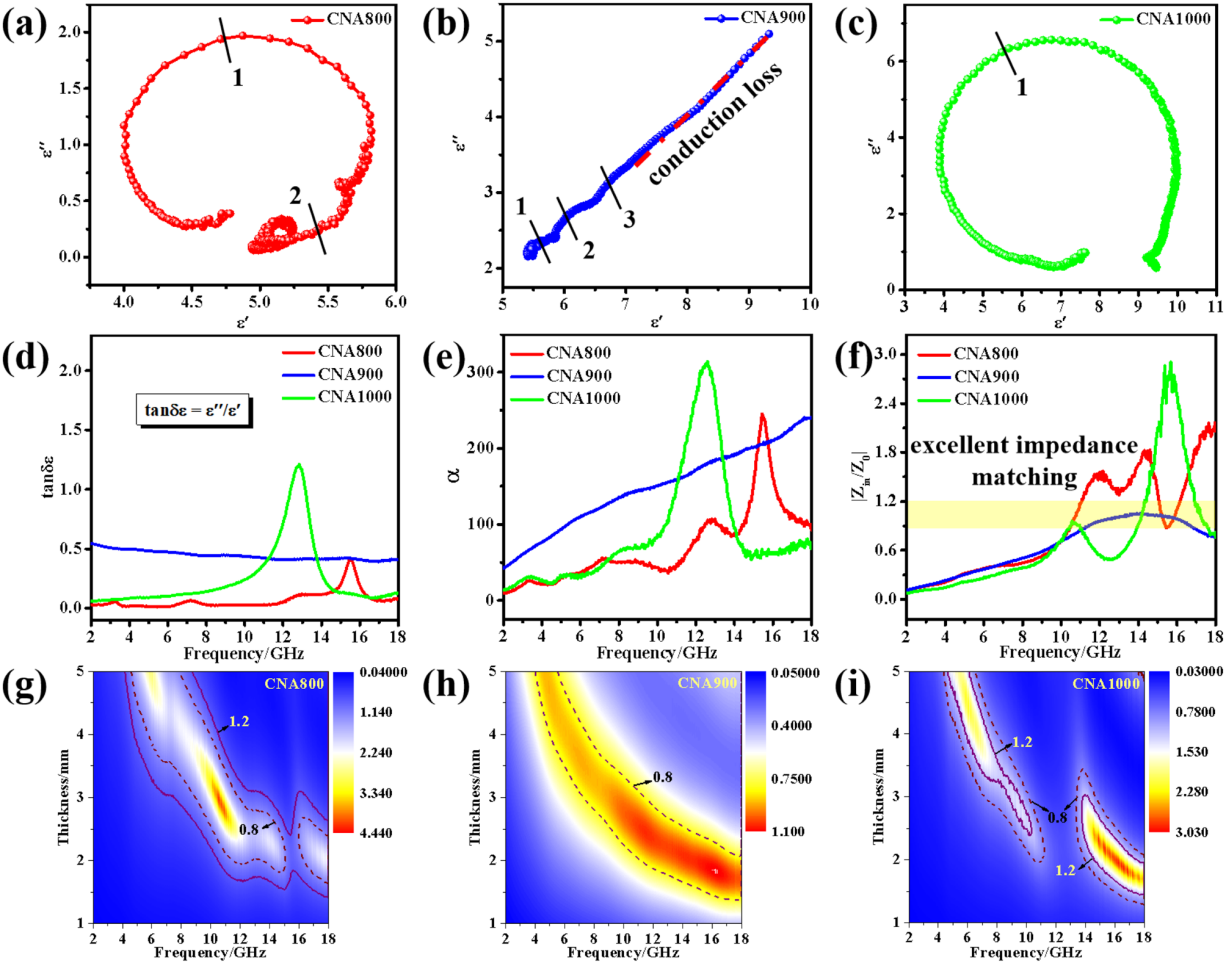
Cole–Cole curves of (**a**) CNA800, (**b**) CNA900 and (**c**) CNA1000. (**d**) Dielectric loss tangent of the as-synthesized samples. (**e**) Attenuation constants and (**f**) comparison of impedance matching at the layer thickness of 2.1 mm of the as-prepared samples. (**f**) 2D representations of *Z* values for (**g**) CNA800, (**h**) CNA900 and (**i**) CNA1000 in the testing frequency range.

According to Havriliak–Negami function and Debye theory, polarization relaxation and conduction loss play significant roles in explaining dielectric loss mechanisms in the whole measuring frequency, which can be described as the following equations^33,34^:

10 $$ \varepsilon_{r} = \, \varepsilon_{\infty } + \left( {\varepsilon_{s} - \varepsilon_{\infty } } \right)/\left( {1 + j2\pi f\tau } \right) = \varepsilon \prime - j\varepsilon \prime \prime $$

11 $$ \varepsilon \prime \, = \, \varepsilon_{\infty } + \left( {\varepsilon_{s} - \varepsilon_{\infty } } \right)/\left( {1 + \left( {2\pi f} \right)^{2} \tau^{2} } \right) $$

12 $$ \varepsilon \prime \prime \, = \, 2\pi f\tau \left( {\varepsilon_{s} - \varepsilon_{\infty } } \right)/\left( {1 + \left( {2\pi f} \right)^{2} \tau^{2} } \right) $$

13 $$ \left( {\varepsilon \prime - \varepsilon_{\infty } } \right)^{2} + \left( {\varepsilon \prime \prime } \right)^{2} = \, \left( {\varepsilon_{s} - \varepsilon_{\infty } } \right)^{2} $$

where *ε*_*∞*_ represents the relative dielectric permittivity at infinite frequency, *ε*_*s*_ is the static permittivity, and *τ* stands for the polarization relaxation time, respectively. Thus, it can be deduced that the *ε′*-*ε″* curves (Cole–Cole plots) should be semicircles. Generally, the dielectric polarization mainly comes from ion polarization, molecular polarization, atomic polarization and electron polarization, which is closely related to microwave frequency^35^. Ionic polarization usually occurs in low frequency range (< 10^5^ Hz), so it cannot play a part in the testing frequency from 2 to 18 GHz. As a matter of fact, the essence of molecular polarization can be regarded as dipole polarization, since molecular polarization comes from the change of dipole moments. Compared with dipole polarization and ionic polarization, atomic polarization and electronic polarization have very limited effect on dielectric loss, so they can be ignored during the frequency range. Thus, in this work, we just talk about the dipole polarization in the testing frequency range. In order to intuitionally observe the semicircles variation trend, Cole–Cole curves are provided in Fig. 5a–c. Obviously, there appear more distorted semicircles for CNA900 than for CNA800 and CNA1000. Namely, CNA900 possesses more Debye relaxation properties than the other two samples, which may be caused by more dipole polarization behaviors of CNA900. Owing to the shrinkage of the size of the as-prepared samples, interfaces between not only CoNi particles but also Co and Ni elements may lead to the formation of abundant dipoles. In addition, the Cole–Cole plot of CNA900 exhibits another straight line, which is related to conduction loss caused by CoNi species^36^. Therefore, for CNA900, both conduction loss and polarization loss are beneficial to dielectric loss. Different from CNA900 sample, CNA800 and CNA1000 samples with fewer semicircles and none straight-line part reveal that conduction loss has a limited effect while polarization relaxation plays a dominant role^37^. This is well corresponding to the resonance peaks (the fluctuation of the *ε′* and *ε″* values at high frequency) that appeared in CNA800 and CNA1000 samples, which can be seen from Fig. 3a,c. As described in Fig. 5d, the average values of dielectric loss tangents (tan *δ*_*ε*_ = ε″/ε′) for CNA800, CNA900 and CNA1000 are 0.07, 0.45, and 0.25. For CNA900, average value of tan *δ*_*ε*_ (0.45) is about twice as high as average value of *tan δ*_*μ*_ (0.21), so it is not hard to conclude that dielectric loss plays a decisive role in enhancing microwave dissipation performance. For the purpose of further understanding the microwave attenuation performance of the as-made samples, attenuation constants *α* can be gained by the following formula^38,39^:

14 $$ \alpha { = }\frac{\sqrt 2 \pi f}{c}\sqrt {\left( {\mu ^{\prime\prime}\varepsilon ^{\prime\prime} - \mu ^{\prime}\varepsilon ^{\prime}} \right) + \sqrt {\left( {\mu ^{\prime\prime}\varepsilon ^{\prime\prime} - \mu ^{\prime}\varepsilon ^{\prime}} \right)^{2} + \left( {\mu ^{\prime}\varepsilon ^{\prime\prime} - \mu ^{\prime\prime}\varepsilon ^{\prime}} \right)^{2} } } $$

Evidently, the calculated average *α* values of CNA800/900/1000 are 70.17, 148.74 and 87.07, respectively, indicating the as-prepared CNA900 show the strongest electromagnetic attenuation ability among all final products (Fig. 5e). Apart from the above factors, the impedance matching property which can be beneficial for microwave to enter into the as-synthesized materials is another factor that needs to be taken into account. The impedance matching property can be described as follows^40^:

15 $$ Z = \, \left| {Z_{in} /Z_{0} } \right| $$

16 $$ Z_{in} = \, \left( {\mu_{r} /\varepsilon_{r} } \right)^{{{1}/{2}}} Z_{0} $$

It should be pointed out that if there exhibits 0 reflection between air and the surface of the microwave absorber (where the value of *Z* is near 1), optimum impedance matching property could be achieved. At the layer thickness of 2.1 mm, effective |*Z*_*in*_*/Z*_*0*_| values (0.8–1.2) of CNA900 cover the working frequency range from 10.68 GHZ to 17.48 GHz (Fig. 5e), and this frequency range is similar to effective bandwidth (where RL < − 10 dB). For comparison, Fig. 5g–i provide 2D representations of the *Z* values for CNAT products, and the values near 1 (0.8–1.2) are marked by purple lines. In detail, the purple dash lines stand for *Z* = 0.8 while full lines signify *Z* = 1.2. Evidently, the *Z* values (from 0.8 to 1.2) of both CNA800 and CNA1000 cover narrower frequency range than that of CNA900, which may be caused by more contact interfaces and larger attenuation ability of CoNi particles. Therefore, preserving suitable impedance matching properties and outstanding attenuation capacity may be an effective way to make CNA900 composite desirable microwave absorbers.

**Figure 6:**
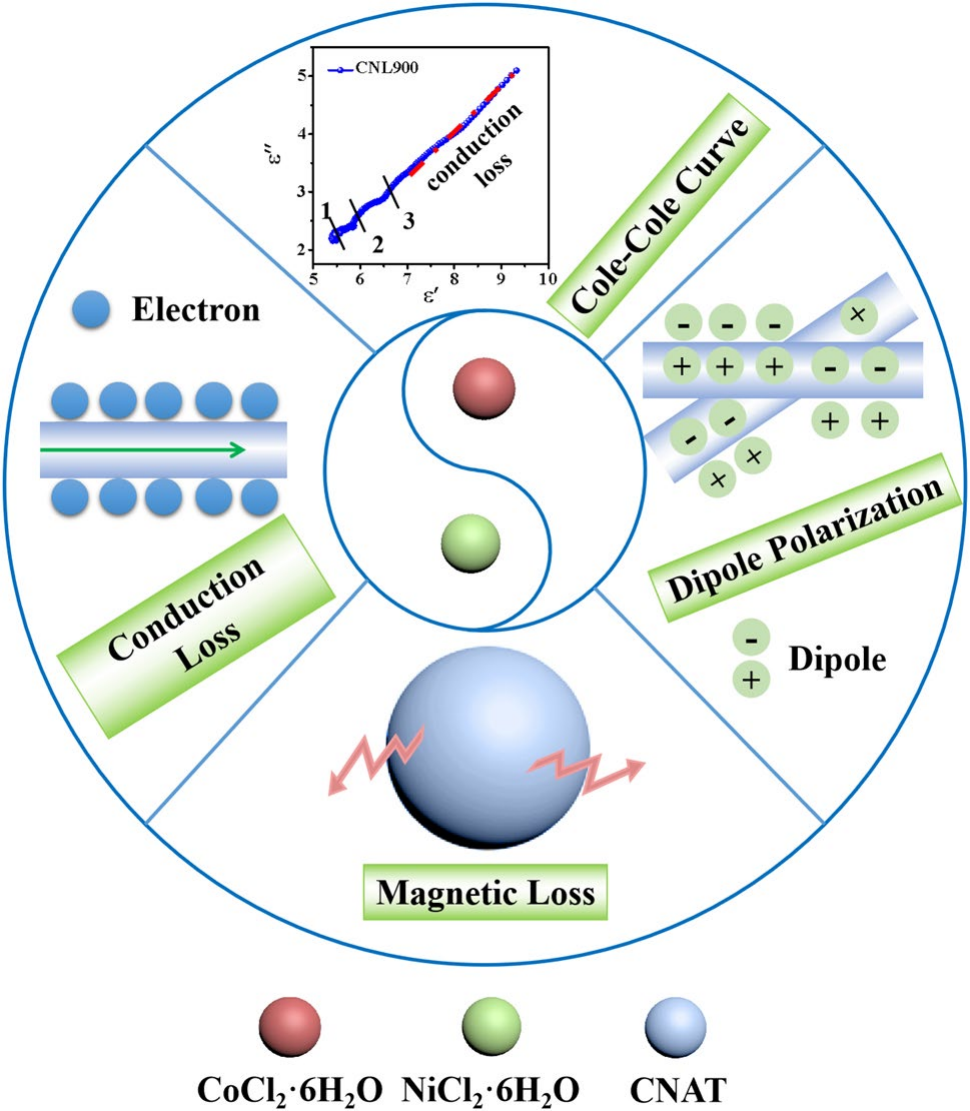
Schematic illustration of microwave absorption mechanisms of the CNAT composites.

According to the previous analysis, the reasons why CNA900 exhibits superb microwave absorption are as follows (Fig. 6): (1) the metal soft magnetic material brings natural resonance, exchange resonance and eddy current loss mechanisms that strengthen the microwave absorption to some extent, (2) the conduction loss induced by conductive bimetallic components that allows more microwave to be attenuated, (3) the dipole polarization caused by migration speed of dipoles are out of step of external electric field can contribute to improve dielectric loss ability, (4) the superior impedance matching performance that allows abundant electromagnetic waves to transmit into the absorption coating layer rather than being reflected. Accordingly, the annealing temperature plays a significant role in forming right-sized particles for achieve enough contact interfaces. For comparison, the crucial indices of relative CoNi-based materials for microwave absorption performance are summarized in Table 1. The relevant scholars indeed have made remarkable contributions in previous works. Nevertheless, the as-prepared CNA900 sample exhibits the minimum reflection loss and the broadest effective frequency bandwidth among all relative materials. Thus, CNA900 in this work is supposed to be a promising candidate for effectively exhaustion and absorption of microwave.

**Table 1:** Microwave absorption performance of relative materials.

Sample	Filling ratio (wt %)	Minimum RL value		RL ≤ − 10 dB			References
		d_m_ (mm)	RL_min_ (dB)	d_m_ (mm)		*f*e (GHz)	
CoNi microspheres	60	2.5	− 41	1.5	4.16		Ref.^41^
CoNi microflowers	20	2	− 28.5	2	6.5		Ref.^42^
Co_67_Ni_33_/Ni_0.6_Zn_0.4_Fe_2_O_4_	60	2.1	− 43.8	2.1	5		Ref.^43^
CoNi-CuO	50	2.5	− 25.1	2.5	3.4		Ref.^44^
CoNi@TiO_2_	30	2.0	− 25	2.0	6.2		Ref.^45^
CoNi@(CoO-NiO)	50	5	− 38.1	2	5.9		Ref.^46^
CoNi@SiO_2_@C	50	2.2	− 46	2.2	5.6		Ref.^47^
Co-Ni-Sn/PANI	25	3.2	− 36.9	3.2	2.46		Ref.^48^
CNA900	55	2.1	− 49.79	2.3	6.84		This work

## Conclusion

In summary, a facial co-precipitation method and an effective calcinations process have been taken to fabricate a series of CNAT composites. The composites obtained at different temperatures present diverse morphology with change in size. Moreover, it also showed excellent microwave absorption performance with strong attenuation abilities and appropriate impedance matching properties. Remarkably, both the working bandwidth and RL_min_ values have been satisfactorily enlarged, the CNA900 sample can achieve the optimum RL value of − 49.79 dB with the matching thickness of merely 2.1 mm and the broadest working frequency of 6.84 GHz at 2.3 mm. Therefore, this work opens a novel pathway for the applications of CNAT materials derived from nanoflower-like CoNi bimetallic hydroxides in high-performance microwave absorbing field.

## Methods

### Materials

Nickel (II) chloride hexahydrate (NiCl_2_·6H_2_O), cobalt (II) chloride hexahydrate (CoCl_2_·6H_2_O) and ammonium hydroxide (NH_3_·H_2_O) were all bought from the Nanjing chemical reagent Co., Ltd. All of the chemicals were analytical pure and employed without further purification.

### Synthesis of CNAT

As depicted in Fig. 1, CNAT composites were fabricated via facile co-precipitation method and calcinations process. In a typical synthesis, 0.71 g of NiCl_2_·6H_2_O along with 0.71 g of CoCl_2_·6H_2_O were dissolved in 60 mL of deionized water and rapidly stirred for 10 min. Then, concentrated ammonium hydroxide was diluted to 1 mol·L^−1^ for the purpose of further utilization. After that, the as-pretreated ammonia hydroxide was added dropwise into the above uniform solution at 50 °C under continuous stirring. After further centrifugal washing (with deionized water and anhydrous ethanol) and blast air drying process, the CoNi bimetallic hydroxides (CoNiBH) can be formed. Finally, CoNi alloys products were gained from pyrolysis of the hydroxide precursors under inert atmosphere. In detail, a certain amount CoNi bimetallic hydroxide powders were put into a combustion boat and annealed at three different temperatures in tube furnace for 2 h under nitrogen atmosphere with a slow heating rate of 2 °C·min^−1^. The corresponding composites were defined as CNAT, where T was the annealing temperature (T = 800, 900, 1000 °C).

### Characterization

The morphology and microstructure were characterized by field emission scanning electron microscopy (FE-SEM, Hitachi S4800) and transmission electron microscopy (TEM, Tecnai G2F30 S-TWIN). The composition and phase of the as-prepared samples were obtained by a Bruker D8 ADVANCE X-ray diffractometer (XRD) using Cu K*α* radiation (*λ* = 1.5604 Å). Electromagnetic parameters were successfully obtained by an Agilent PNA N5244A vector network analyzer (VNA). The coaxial rings (φ_out_ of 7.00 mm and φ_in_ of 3.04 mm) for testing were prepared by mixing 55 wt % CNAT power with 45 wt % paraffin wax matrix. Vibrating sample magnetometer (VSM, Lakeshore, Model 7400 series) was used to form the hysteresis loops of the specimens at an external magnetic field of 10,000 Oe.

## References

[1] Lv H, Yang Z, Wang P, Ji G, Song J, Zheng L, Zeng H, Xu Z (2018). A voltage-boosting strategy enabling a low-frequency, flexible electromagnetic wave absorption device. Adv. Mater..

[2] Weng G (2018). Layer-by-layer assembly of cross-functional semi-transparent MXene-carbon nanotubes composite films for next-generation electromagnetic interference shielding. Adv. Funct. Mater..

[3] You W (2017). Dipolar-distribution cavity γ-Fe_2_O_3_@C@α-MnO_2_ nanospindle with broadened microwave absorption bandwidth by chemically etching. Small.

[4] Quan B (2017). Strong electromagnetic wave response derived from the construction of dielectric/magnetic media heterostructure and multiple interfaces. ACS Appl. Mater. Interfaces.

[5] Liu Q (2016). CoNi@SiO_2_@TiO_2_ and CoNi@Air@TiO_2_ microspheres with strong wideband microwave absorption. Adv. Mater..

[6] Trukhanov SV (2017). Investigation into the structural features and microwave absorption of doped barium hexaferrites. Dalton Trans..

[7] Huang Z (2018). Ultra-broadband wide-angle terahertz absorption properties of 3D graphene foam. Adv. Funct. Mater..

[8] Lv J (2019). Nanofiber network with adjustable nanostructure controlled by PVP content for an excellent microwave absorption. Sci. Rep..

[9] Liu J (2018). Multifunctional, superelastic, and lightweight MXene/polyimide aerogels. Small.

[10] Qu XH (2020). Nitrogen-doped graphene layer-encapsulated NiFe bimetallic nanoparticles synthesized by an arc discharge method for a highly efficient microwave absorber. Inorg. Chem. Front..

[11] Almasi-Kashi M (2018). Improvement of the microwave absorption properties in FeNi/PANI nanocomposites fabricated with different structures. J. Alloys Compd..

[12] Xu DM (2020). Bimetal oxide-derived flower-like heterogeneous Co/MnO@C composites with synergistic magnetic-dielectric attenuation for electromagnetic wave absorption. J. Mater. Chem. C.

[13] Wang X (2019). Stamen-petal-like CeO_2_/NiMn layered double hydroxides composite for high-rate-performance supercapacitor. J. Alloys Compd..

[14] Song F (2014). Exfoliation of layered double hydroxides for enhanced oxygen evolution catalysis. Nat. Commun..

[15] Margarita D (2005). Bio-nanocomposites based on layered double hydroxides. Chem. Mater..

[16] Sun Z (2018). Amorphous boron oxide coated NiCo layered double hydroxide nanoarrays for highly efficient oxygen evolution reaction. ACS Sustain. Chem. Eng..

[17] Bai S (2018). Fabricating of Fe_2_O_3_/BiVO_4_ heterojunction based photoanode modified with NiFe-LDH nanosheets for efficient solar water splitting. Chem. Eng. J..

[18] Li X (2017). Nanoflakes of Ni-Co LDH and Bi_2_O_3_ assembled in 3D carbon fiber network for high-performance aqueous rechargeable Ni/Bi battery. ACS Appl. Mater. Interfaces.

[19] Wang XK (2020). Excellent electromagnetic wave absorption properties of porous core-shell CoO/Co@C nanocomposites derived from a needle-shaped Co(OH)_2_@ZIF-67 template. J. Alloys Compd..

[20] Yao GD (2012). Direct and highly efficient reduction of NiO into Ni with cellulose under hydrothermal conditions. Ind. Eng. Chem. Res..

[21] Gu W (2020). Multifunctional bulk hybrid foam for infrared stealth, thermal insulation, and microwave absorption. ACS Appl. Mater. Interfaces.

[22] Liang X (2020). The environment-stable Co_x_Ni_y_ encapsulation in stacked porous carbon nanosheets for enhanced microwave absorption. Nano-Micro Letter..

[23] Yang Z (2016). Rational construction of graphene oxide with MOF-derived porous NiFe@C nanocubes for high-performance microwave attenuation. Nano Res..

[24] Zhang X (2018). Tunable high-performance microwave absorption of Co_1-x_S hollow spheres constructed by nanosheets within ultralow filler loading. Adv. Funct. Mater..

[25] Zhang Z (2019). A biomass derived porous carbon for broadband and lightweight microwave absorption. Sci. Rep..

[26] Zhang X (2015). Thermal conversion of an Fe_3_O_4_@metal-organic framework: A new method for an efficient Fe-Co/nanoporous carbon microwave absorbing material. Nanoscale.

[27] Yang Z (2019). Hierarchical formation mechanism of anisotropic magnetite microflakes and their superior microwave attenuation properties. J. Alloys Compd..

[28] Savilov SV (2018). 3D Frameworks with variable magnetic and electrical features from sintered cobalt-modified carbon nanotubes. ACS Appl. Mater. Interfaces.

[29] Wang Y (2019). Enhanced microwave absorption performances of polyaniline/graphene aerogel by covalent bonding. Compos. Part B Eng..

[30] Liu W (2018). A versatile route toward the electromagnetic functionalization of metal-organic framework-derived three-dimensional nanoporous carbon composites. ACS Appl. Mater. Interfaces.

[31] An Z (2009). Facile preparation and electromagnetic properties of core-shell composite spheres composed of aloe-like nickel flowers assembled on hollow glass spheres. J. Phys. Chem. C.

[32] Wang J (2015). Enhanced microwave absorption properties of epoxy composites reinforced with Fe_50_Ni_50_-functionalized grapheme. J. Alloys Compd..

[33] Wang H (2019). Interface modulating CNTs@PANi hybrids by controlled unzipping of the walls of CNTs to achieve tunable high-performance microwave absorption. ACS Appl. Mater. Interfaces.

[34] Liang XH (2019). Self-assembly three-dimensional porous carbon networks for efficient dielectric attenuation. ACS Appl. Mater. Interfaces.

[35] Quan B (2019). Defect engineering in two common types of dielectric materials for electromagnetic absorption applications. Adv. Funct. Mater..

[36] Cheng Y (2020). A flexible and lightweight biomass-reinforced microwave absorber. Nano-Micro Lett..

[37] Xu DM (2019). Facile synthesis of three-dimensional porous Co/MnO composites derived from bimetal oxides for highly efficient electromagnetic wave absorption. ACS Sustain. Chem. Eng..

[38] Zhou P (2020). Silica-modified ordered mesoporous carbon for optimized impedance-matching characteristic enabling lightweight and effective microwave absorbers. ACS Appl. Mater. Interfaces.

[39] Lou Z (2019). Phenolic foam-derived magnetic carbon foams (MCFs) with tunable electromagnetic wave absorption behavior. Chem. Eng. J..

[40] Yang L (2020). Multiple polarization effect of shell evolution on hierarchical hollow C@MnO_2_ composites and their wideband electromagnetic wave absorption properties. Chem. Eng. J..

[41] Wang ZZ (2019). Ferromagnetic and excellent microwave absorbing properties of CoNi microspheres and heterogeneous Co/Ni nanocrystallines. RSC Adv..

[42] Liu QH (2015). Insights into size-dominant magnetic microwave absorption properties of CoNi microflowers via off-axis electron holography. ACS Appl. Mater. Interfaces.

[43] Wang, M. et al. Single-layer and double-layer microwave absorbers based on Co_67_Ni_33_ microspheres and Ni_0.6_Zn_0.4_Fe_2_O_4_ nanocrystals. *J. Magn. Magn. Mater.* **425** 25–30 (2017).

[44] Gao SS (2017). Facile solvothermal synthesis of novel hetero-structured CoNi-CuO composites with excellent microwave absorption performance. RSC Adv..

[45] Chen C (2016). Fabrication of hierarchical TiO_2_ coated Co_20_Ni_80_ particles with tunable core sizes as high-performance wide-band microwave absorbers. Phys. Chem. Chem. Phys..

[46] Ni C (2020). Microwave absorption properties of microporous CoNi@(NiO-CoO) nanoparticles through dealloying. J. Magn. Magn. Mater..

[47] Zhou SH (2018). Synthesis and microwave absorption enhancement of CoNi@SiO_2_@C hierarchical structures. Ind. Eng. Chem. Res..

[48] Almasi-Kashi M (2019). The role of Sn, Zn, and Cu additions on the microwave absorption properties of Co-Ni alloy nanoparticles. Mater. Res. Bull..

